# Nurse-supported self-monitoring of serum urate by gout patients using a treat-to-target approach: a feasibility study

**DOI:** 10.1093/rap/rkag064

**Published:** 2026-06-05

**Authors:** Jeffrey van der Ven, Marcel Flendrie, Anouk Brokking, Noortje van Herwaarden, Bart J F van den Bemt, Lise M Verhoef

**Affiliations:** Department of Research, Sint Maartenskliniek, Ubbergen, The Netherlands; Radboud University Medical Centre, Nijmegen, The Netherlands; Department of Rheumatology, Sint Maartenskliniek, Ubbergen, The Netherlands; Radboud University, Nijmegen, The Netherlands; Department of Rheumatology, Sint Maartenskliniek, Ubbergen, The Netherlands; Department of Pharmacy, Pharmacology and Toxicology, Radboud University Medical Centre, Nijmegen, The Netherlands; Department of Research, Sint Maartenskliniek, Ubbergen, The Netherlands; Department of Pharmacy, Pharmacology and Toxicology, Radboud University Medical Centre, Nijmegen, The Netherlands; Department of Research, Sint Maartenskliniek, Ubbergen, The Netherlands

**Keywords:** gout, feasibility, nursing, eHealth, self-management, urate-lowering-therapy, point-of-care testing

## Abstract

**Objective:**

To assess the feasibility of a nurse-supported, treat-to-target (T2T) self-monitoring approach for gout patients initiating urate-lowering therapy (ULT) using point-of-care testing (POCT) of serum urate (SU) levels.

**Methods:**

A 24-week prospective feasibility study was conducted at the Sint Maartenskliniek in the Netherlands among 32 patients starting ULT, or recently started ULT and not at target. Participants received a POCT device to self-measure SU levels at home and report results every 4 weeks via a digital platform. Nurses provided education and dosing advice based on SU levels and monitored patient follow-up. Feasibility was assessed using Bowen’s framework. Patient data were obtained through questionnaires and chart review, and stakeholder perspectives were collected through semi-structured interviews.

**Results:**

Patients found the intervention highly acceptable. Adherence to SU measurements was 93%, with minimal burden and few problems reported. The majority of the patients reached their SU target (75%). Patients had a median of 2 gout flares (interquartile range [IQR] 0–3). Stakeholders valued the intervention for the potential to increase patient engagement and care efficiency, though they emphasized the need for evidence on cost-effectiveness, clear protocols and appropriate patient selection.

**Conclusion:**

Nurse-supported self-monitoring for gout was acceptable, feasible and practical in this selected group of gout patients. The short-term SU target attainment was encouraging, warranting larger comparative randomized studies with longer follow-up to establish (cost-) effectiveness, and long-term disease control.

Key messages

- Nurse-supported serum urate self-monitoring is a feasible and patient-accepted treat-to-target strategy for gout.
- This uncontrolled intervention achieved high measurement adherence and 75% serum urate target attainment within 24 weeks in a selected group of patients.
- Successful implementation requires clear protocols, digital integration and evidence on (cost-) effectiveness and reimbursement.

## Introduction

Gout is the most common form of inflammatory arthritis [1]. It typically presents as acute flares characterized by severe joint pain, swelling, redness and warmth in one or more joints and may progress to chronic, destructive arthropathy. The prevalence of gout varies between populations, ranging from less than 1% to 6.8% [2]. Acute gout results from the accumulation of monosodium urate crystals in the joints, typically due to chronic hyperuricemia. Effective management is possible through urate-lowering therapy (ULT) [3, 4]. A treat-to-target (T2T) approach, in which the dose of ULT is up-titrated until a serum urate (SU) target below 0.36 mmol/l (6 mg/dl) is reached, will prevent the occurrence of future gout flares, tophi and increase quality of life [3, 4]. In patients with more severe gout and high urate crystal burden, a lower SU target, below 0.30 mmol/l (5 mg/dl) is advocated [5]. However, only one-third of ULT-treated patients reach these targets, resulting in unnecessary gout flares [6–10].

Nurse-led T2T care can improve SU target attainment to over 90% and reduces gout flares [7, 11, 12]. However, it is resource-intensive and challenged by staff shortages [13]. The increasing prevalence of gout and the resulting strain on healthcare professionals (HCPs) necessitate innovative care strategies.eHealth can enhance gout care through remote education and monitoring, which has the potential to optimize workforce utilization. Self-monitoring of SU levels, using point-of-care testing (POCT) might enable patient involvement, while reducing laboratory testing and consultations [8]. POCT-devices have shown validity for measuring SU [14, 15], and studies show patient-led home monitoring of SU improves urate control, reduces gout flares and may be cost-effective [8, 16, 17]. Patients find this approach useful and convenient, offering better control of their disease [16].

Based on current literature, a nurse-supported T2T intervention with POCT self-monitoring and remote support may improve patient involvement, execution of the T2T strategy and care efficiency compared with usual rheumatologist-led care.

However, before conducting large scale trials, it is important to study the feasibility of this approach, and identify barriers and facilitators for successful implementation in real-world settings. This research therefore aims to assess the feasibility of patient self-monitoring SU using POCT and a nurse-supported T2T approach for gout patients initiating ULT in secondary care, and explore short-term treatment outcomes.

## Methods

### Study design and setting

This 24-week prospective single-centre feasibility study was performed from May 2024 to April 2025 at the Sint Maartenskliniek in Nijmegen, The Netherlands. Bowen’s feasibility framework was used to guide data collection [18]. The included focus areas of this framework are acceptability, demand, implementation, integration, practicality and limited-efficacy (including clinical outcomes, healthcare utilization and patient-reported outcome measures [PROMs]). Adaptation and expansion were deemed to be not applicable in the current context. Patients and stakeholders (involved care providers and other staff) were included in this study. The CONSORT-extension checklist was used to ensure quality and transparency of reporting (Supplementary Data S1, available at *Rheumatology Advances in Practice* online). Patient information, questionnaires and study procedures were reviewed by two patient research partners, providing input on appropriateness and comprehensibility of the questions and any other concerns from the patients’ perspective.

### Recruitment and participants

In this study, patients, HCPs and hospital management participated. Inclusion criteria for patients were: clinical diagnosis of gout, age ≥16, initiating or recently started ULT (≤6 months) and not at SU target (<0.36 mmol/l [<6 mg/dl], or <0.30 mmol/l [<5 mg/dl] in case of tophaceous gout, chronic arthropathy or frequent attacks) based on lab results within the last 6 weeks, able to provide informed consent, access to a digital device with internet and email, willingness to use a POCT at home, and proficient in Dutch. Exclusion criteria were: contraindications for ULT, inability to measure SU at home, severe kidney disease (Chronic Kidney Disease Epidemiology Collaboration (CKD-EPI) <0.45 ml/min/1.73 m^2^) or severe liver damage/dysfunction (alanine-aminotransferase (ALAT) >3× upper limit of normal or a history of cirrhosis). Consecutive patients visiting the outpatient clinic were screened for eligibility. If eligible, the treating rheumatologists discussed the possibility of study participation with the patient and referred those interested to the study team for information and consent. For this feasibility study, no formal sample size calculation was performed; instead, a general recommendation of 30 participants for feasibility studies was followed [19]. Therefore, recruitment continued until at least 30 patients had used the intervention for 12 weeks. We included a variety of stakeholders involved in the study’s execution or management. Stakeholders received study information and were invited to participate in interviews. Before each interview, verbal informed consent was obtained after explaining the study’s purpose and data handling procedures.

### Intervention for patients

Participating patients received education on the POCT device (Wellion LUNA Trio, MED TRUST Handelsges.m.b.H., Marz, Austria) through an instruction video, device manual, and, if needed, face-to-face instruction by a doctor’s assistant. Patients also received digital written information on the self-monitoring process, digital communicating SU levels, treatment goals, contact details of healthcare providers and a troubleshooting guide.

Patients were asked to report SU levels every 4 weeks for 24 weeks via an app-based survey through the hospital’s patient portal. The survey also collected data on side effects, questions, remarks and gout flares, using Gaffo et al.’s [20] gout flare definition.

As part of usual care, patients received standardized written educational material about gout and ULT from the treating physician. Additional education was provided in a face-to-face consultation by a specialized rheumatology nurse.

### Intervention deliverance by healthcare providers

Patients were monitored by rheumatology nurses (nurses specialized in care for patients with rheumatic diseases) under supervision of a rheumatologist (M.F.). Nurses provided ULT dosage advice, based on SU levels, via a personal text message posted on the patients’ portal feed. They also provided a 1-h consultation within 8 weeks after start of the study to provide education on gout, medication and lifestyle, unless already done as part of the local usual care treatment protocol. At week 5, patients received a 15-min follow-up call to discuss progress and engagement. Email support by the nurses was available throughout the study period. Treatment followed the T2T approach with dose adjustments guided by 4-weekly home SU measurements, aiming for SU targets of <0.36 mmol/l, or < 0.30 mmol/l in cases of tophaceous gout, chronic arthropathy or frequent gout flares. Allopurinol was the first-line treatment (up-titrated to a maximum of 900 mg/day). Febuxostat (up to 120 mg/day) or benzbromarone (up to 200 mg/day) were alternatives in case of intolerance or inability to reach SU targets.

Treating rheumatologists reviewed laboratory safety test results (CKD-EPI, blood counts and alanine aminotransferase [ALT]), performed at week 8 and 24. The intervention did not include follow-up outpatient visits.

### Patient data collection

Patient data were collected through questionnaires (using CastorEDC, at baseline, 12 weeks and 24 weeks), the 4-weekly app survey and chart review. Patient characteristics (age, sex, disease duration, severity and comorbidities) were extracted from electronic health records (EHRs). The baseline questionnaire assessed sociodemographics, health literacy (HLSF-12) [21], digital skills (Pharos Quickscan) [22] and prior experience with the patient portal and video consultations.

Feasibility for patients was assessed across four domains of the Bowen framework (excluding integration and implementation): acceptability, demand, practicality and clinical outcomes. Full overview of items and instruments is provided in Supplementary Data S2, available at *Rheumatology Advances in Practice* online.

*Acceptability* was assessed at weeks 12 and 24 using the Dutch system usability scale (SUS) [23] (range: 0–100), Likert-scale questions on perceived usefulness, satisfaction with and appropriateness of the POCT-device and perceived mental and physical burden.

*Demand* was measured through patient-reported intention and actual use of the device, using three 5-point Likert scale items at 12 and 24 weeks. Mean measurement adherence per patient was calculated as the percentage of completed SU measurements over scheduled measurements, and the number of reminders sent (email/app and telephone) was recorded.

*Practicality* was assessed at weeks 12 and 24 using four 5-point Likert scale questions on the use of the POCT-device and digital communication of SU values (Supplementary Data S2, available at *Rheumatology Advances in Practice* online). Issues with the POCT-device, reported through questionnaires and nurse communications, were recorded, along with the number of patients requiring face-to-face instruction. Reasons for withdrawal were collected and reported.

*Limited efficacy (clinical outcomes, healthcare utilization and PROMs):* pre-inclusion, baseline and final SU values were obtained from EHRs and 4-weekly self-monitoring app surveys. Baseline and 24-week ULT usage and dosage, as well as rheumatologist visit frequency, duration and reasons, were extracted from patient records, along with nurse visit counts and time spent. Self-reported gout flare rates came from the app surveys. Patient-reported outcomes (PROMs), patient activation (Patient Activation Measure-13 (PAM-13)), quality of life (EuroQol 5-Dimension 5-Level (EQ-5D-5L)) [24] and medication beliefs (Beliefs about Medicines Questionnaire (BMQ)) [25] were assessed at baseline and 24 weeks.

### Stakeholders data collection

Stakeholders’ perspectives on acceptability, demand, practicality, implementation and integration, as outlined by Bowen’s feasibility framework [18], of nurse-supported self-monitoring of SU were assessed through semi-structured interviews (30–60 min). Stakeholders consisted of rheumatology nurses, rheumatologists and outpatient clinic management. The interview guide was constructed based on Bowen’s feasibility framework and was pilot-tested with a rheumatologist involved in the study. The interview guide was refined throughout the process, and further tailored to the role and expertise of each interviewee. The final interview guide is presented in Supplementary Data S2, available at *Rheumatology Advances in Practice* online.

### Data analysis

Quantitative data were analysed descriptively using RStudio. Means and S.D. were reported for normally distributed continuous variables, while medians and interquartile ranges (IQRs) were used for non-normal data. Categorical variables were presented as counts and percentages. For the final SU self-measurements, last observation carried forward was used to replace missing SU-values at week 24. Responses on intervention usefulness, perceived burden and patient ability were visualized with bar charts using ggplot2.

EQ-5D-5L index scores were calculated via a crosswalk index calculator with the Dutch value set, ranging from 0.59 to 1.00 (1.00 = best health). From the BMQ, the necessity–concern difference (NCD) was computed by subtracting the concern score from the necessity score. Using median cut-offs on BMQ subscales, four attitudinal profiles were classified: acceptant, ambivalent, sceptic and indifferent [26].

Stakeholder interviews were transcribed verbatim and thematically analysed by two researchers (J.v.d.V. and A.B.) and reviewed by a third researcher (L.M.V.) using an inductive approach in ATLAS.ti (v25), following Braun and Clarke [27]. The researchers independently generated open codes after familiarization, then discussed to reach consensus. Agreed codes were grouped into subthemes, refined through discussion and ultimately organized into main themes by consensus. Illustrative quotes from participants are included in the results to support the themes.

### Ethical considerations

Patients were approached by their rheumatologist and granted permission to be contacted by the research team. All participants provided written informed consent after being informed verbally and in writing and having the opportunity to ask questions. Patient data were pseudonymized to protect privacy. Ethical approval was granted by the Medical Research Ethics Committee of Eastern Netherlands (File Number: 2024–17033). The study adhered to the Declaration of Helsinki and the Dutch Medical Research Involving Human Subjects Act (Wet Medisch-wetenschappelijk Onderzoek met Mensen (WMO)), with data handled in compliance with the Dutch General Data Protection Regulation.

## Results

**Figure 1:**
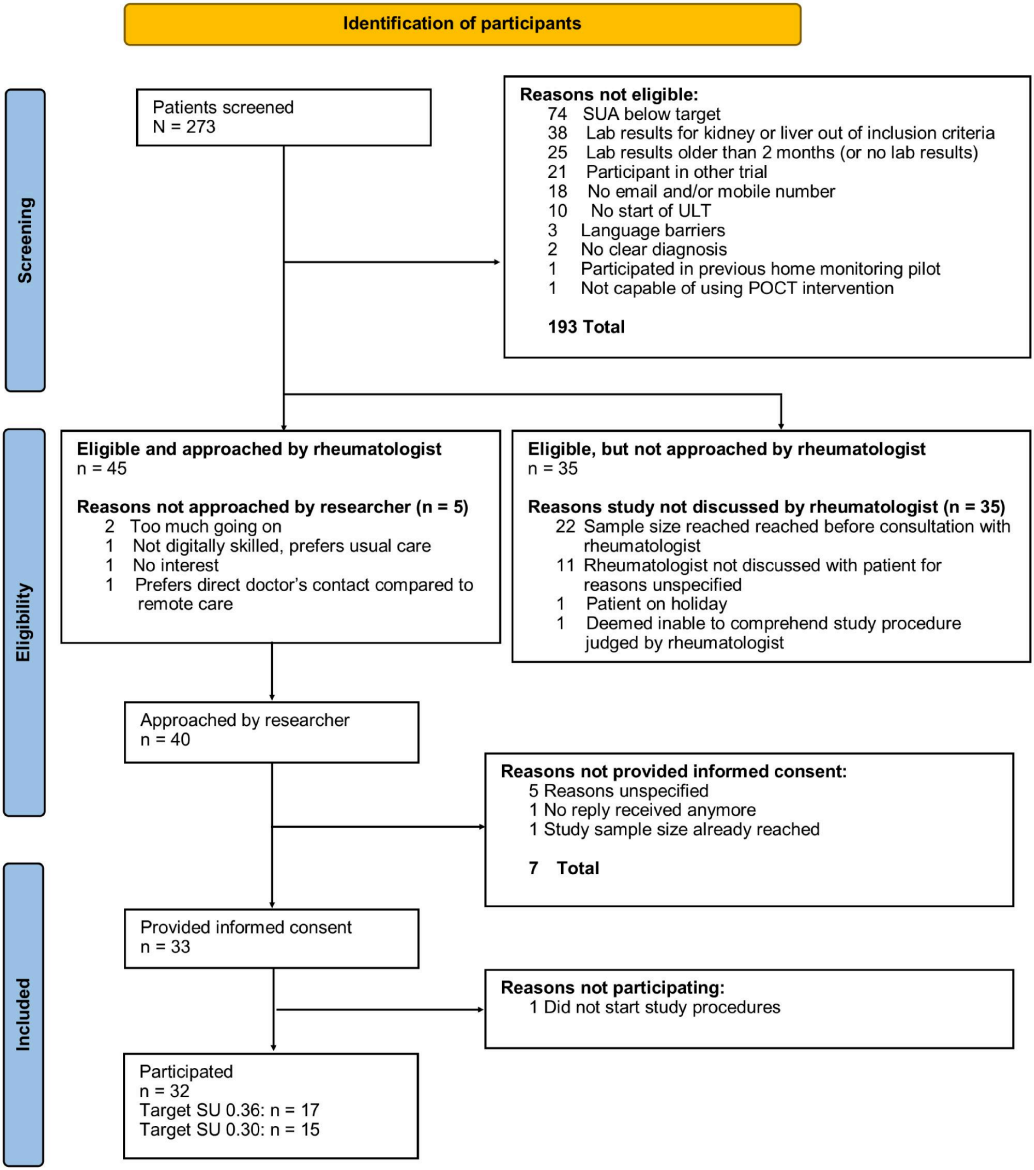
PRISMA flow diagram. Adapted from: Page MJ, et al. The PRISMA 2020 statement: an updated guideline for reporting systematic reviews. BMJ. 2021;372:n71. The process of participant identification, recruitment and inclusion

Of the 273 patients screened, 80 met the eligibility criteria. Of these, 40 were approached to participate in the study, (and 33 patients provided consent, of which one did not start the study procedures due to personal reasons, resulting in 32 participants Fig. 1). One participant was lost to follow-up after the third measurement, available data were included in the analyses. Participant baseline characteristics are shown in Table 1. All participants owned a digital device and patients independently used digital technologies (Supplementary Material 3, Table S1, available at *Rheumatology Advances in Practice* online).

**Table 1:** Characteristics of the study population (*N* = 32).

Characteristics	Values
Mean age (years), S.D. (*N* = 32)	52.9 (14.5)
Sex, *n* (%)	
Male	30 (94)
Female	2 (6)
Marital status, *n* (%)	
Married	25 (78)
Never married	7 (22)
Divorced/widowed	0 0
Level of education,^a^ *n* (%)	
Low	5 (16)
Medium	11 (34)
High	16 (50)
Employment status, *n* (%)	
Studying	1 (3)
Employed	21 (66)
Retired	6 (19)
Fulltime housewife/husband	2 (6)
Unfit for work	2 (6)
Median disease duration,^b^ (months) (IQR)	2.9 (0.8–14.1)
Disease severity, *n* (%)	
Monoarthritis	5 (16)
Oligoarthritis	21 (66)
Polyarthritis	6 (19)
Tophi present, *n* (%)	6 (19)
Erosions present, *n* (%)	7 (22)
Comorbidities related to gout,^c^ *n* (%)	
Cardiovascular disease	8 (25)
Renal disease	5 (16)
Diabetes mellitus	2 (6)
Obesity	5 (16)
Hypertension	12 (38)
None	15 (47)
Health literacy SF12 index,^d^ mean (S.D.)	36.5 (6.2)

a Level of education: low—lower vocational training or below; medium—up to and completion of secondary vocational training; high—higher vocational training and university.

b Disease duration is defined as the time from first gout flare to baseline.

c More than one comorbidity possible.

d The health literacy index ranges from 0 to 50, with 50 indicating the highest health literacy.

### Patient acceptability

**Figure 2:**
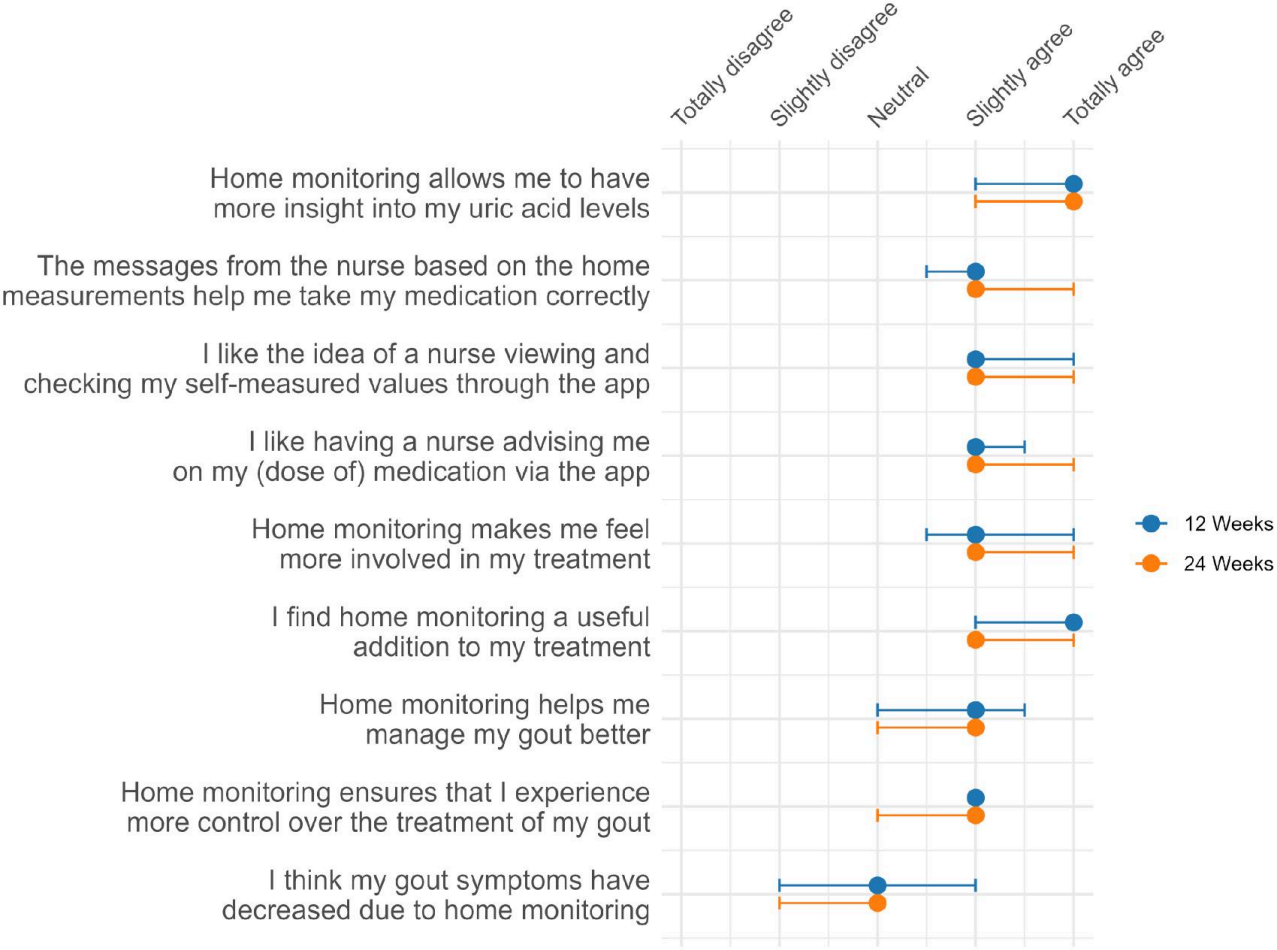
Perceived usefulness. Results of the perceived usefulness statements at week 12 (*n* = 31), and at week 24 (*n* = 29). Dots represent the median on a 5-point Likert scale, and bars indicate the first and third quartile

Based on the perceived usefulness statements, patients found the intervention useful, citing improved insight, added treatment value and greater control over treatment, while remaining neutral about the impact on gout symptoms (Fig. 2).

The median SUS score was 78 (IQR: 70–89) at 12 weeks (*n* = 31) and 85 (IQR: 70–93) at 24 weeks (*n* = 29). All respondents (100%, *n* = 29/29) at week 24 would recommend the intervention. At week 24, the proportion of three-star ratings was 3% (*n* = 1/29), four-star ratings 69% (*n* = 20/29) and five-star ratings 28% (*n* = 8/29) (Supplementary Material 3, Table S2, available at *Rheumatology Advances in Practice* online). Patients reported that self-pricking was neither physically burdensome nor time-consuming, and they felt comfortable both during the home monitoring week and when digitally submitting their values (Supplemental Material 3, Fig. S1, available at *Rheumatology Advances in Practice* online). At week 24, the majority of respondents (69%, *n* = 20/29) rated the devices attractiveness as neutral (Supplementary Material 3, Table S3, available at *Rheumatology Advances in Practice* online), while the rest found the device attractive to use. At week 24, 72% (*n* = 23/29) of respondents regarded the POCT-device as appropriate, 16% (*n* = 5/29) of patients rated the POCT-device as acceptable and 3% (*n* = 1/29) as inappropriate (Supplementary Material 3, Table S4, available at *Rheumatology Advances in Practice* online).

### Patient demand

#### Intention to use

The large majority of respondents (97%, *n* = 28/29) agreed that they would like to use the intervention in the future at week 24 (Supplementary Material 3, Table S5, available at *Rheumatology Advances in Practice* online). Most respondents preferred to use the intervention with the same frequency at week 24 (52%, *n* = 15/29), while 38% (*n* = 11/29) indicated they would use it more often. Willingness to pay for the intervention varied, with 52% (*n* = 15/29) agreeing, 35% (*n* = 10/29) neutral and 14% (*n* = 4/29) disagreeing (Supplementary Material 3, Table S5, available at *Rheumatology Advances in Practice* online).

#### Actual use

In total, 223 measurements were scheduled. To support adherence, 61 reminders were sent through the application to 21 patients. Among these, the median number of reminders per patient during the 24-week period was 2 (IQR: 1–5). Additionally, seven telephone reminders were made for six patients. Despite these efforts, 16 measurements were missed, involving eight patients, with a median of two missing measurements per patient (IQR: 1–2.5). This led to a mean adherence to scheduled measurements of 93%. Reported reasons for missed measurements included forgetting to perform the finger-prick and a lack of testing strips.

### Patient practicality

Patients reported that the instructional video and the explanation of the POCT-device by the doctor’s assistant facilitated the self-monitoring procedure. In addition, patients could easily access support to have their questions addressed in case of any problems (Supplemental Material 3, Fig. S2, available at *Rheumatology Advances in Practice* online). Three patients required in-person POCT instruction and five patients reported issues with their POCT device, prompting replacement; whether due to device malfunction or user error is unclear. Additional materials (test strips and needles) were supplied to five patients. Additional information on patient practicality can be found in Supplementary Material 3, Text S1, available at *Rheumatology Advances in Practice* online.

### Limited efficacy (clinical outcomes, healthcare utilization and PROMs)

SU baseline values and outcomes are presented in Table 2. At week 24, 24 of 32 (75%) reached SU target, while 28 of 32 (88%) reached SU target at least once during follow-up (median no. measurements on target 3 (IQR 2–4)). Patients had a median of 2 (IQR: 0–3) and a mean of 2 (S.D.: 1.8) gout flares over the 6-month study period (Supplementary Material 3, Fig. S3, available at *Rheumatology Advances in Practice* online). Detailed dosing information is presented in Supplementary Material 3, Table S6, available at *Rheumatology Advances in Practice* online. Supplementary Material 3, Table S7, available at *Rheumatology Advances in Practice* online, shows information on adverse events and side-effects.

**Table 2:** Clinical outcomes by SU target group.

	SU before inclusion (mmol/l)	SU baseline POCT (mmol/l)	SU final POCT (mmol/l)	On target final POCT (%)
0.36 mmol/l, *N* = 17, mean (S.D.)	0.46 (0.06)	0.43 (0.08)	0.31 (0.03)	94
0.30 mmol/l, *N* = 15, mean (S.D.)	0.47 (0.10)	0.36 (0.10)	0.31 (0.06)	53

Mean SU levels (S.D.) based on the laboratory measurement prior to inclusion, as well as POCT measurements at baseline and at week 24, are presented. POCT: point-of-care testing; SU: serum urate.

During the study, the median number of rheumatologist visits was 0 (IQR: 0–1), with 19 total visits among 13 patient (Supplementary Material 3, Text S2, available at *Rheumatology Advances in Practice* online). Of these 19 visits, 13 were scheduled with a specific medical reason such as adverse events (reflecting a mean of 6 min [S.D. 12] per patient) while six visits were pre-planned follow-up visits without indication, not in line with the study protocol. A total of 33 questions were forwarded by nurses to rheumatologists, originating from both patients and the nurses themselves. (Supplementary Material 3, Text S2, available at *Rheumatology Advances in Practice* online). Regarding nurse contact, 88% of patients (*n* = 28) had the 1-h nurse visit (5 before and 23 after study initiation). The median number of nurse contacts was 3 (IQR: 2–5), and the mean total time spent with a nurse was 88 min per patient (S.D. 29.3), including the scheduled 1-h visit and the 15-min week 5 follow-up call. The results concerning quality of life, patient activation and beliefs about medication are presented in Table 3.

**Table 3:** Patient activation, quality of life and beliefs about medication.

Variable^∗^	Baseline	Week 24	Change over time
EQ-5D-5L, median (IQR)	0.86 (0.79 to 0.87), *n* = 32	0.83 (0.78 to 1.00), *n* = 29	0.00 (−0.01 to 0.06), *n* = 29
PAM score, median (IQR)	53.20 (47.95 to 59.35), *n* = 31	54.40 (53.20 to 70.20), *n* = 28	2.75 (−1.72 to 9.70), *n* = 28
BMQ			
Necessity score, mean (S.D.)	16.25 (4.25), *n* = 32	16.57 (3.98), *n* = 28	0.79 (2.9), *n* = 28
Concern score, mean (S.D.)	12.09 (3.32), *n* = 32	12.93 (2.1), *n* = 29	0.52 (2.85), *n* = 29
Overuse score, mean (S.D.)	10.78 (2.27), *n* = 32	10.41 (2.44), *n* = 29	−0.45 (2.21), *n* = 29
Harm score, mean (S.D.)	8.66 (2.27), *n* = 32	9.03 (1.84), *n* = 29	0.38 (1.7), *n* = 29
Attitudinal profiles,^a^^,^^b^ *n* (%)			
Acceptant	9 (28)	4 (14)	
Ambivalent	6 (18.8)	12 (43)	
Sceptic	8 (25)	5 (18)	
Indifferent	9 (28)	7 (25)	
NCD score,^c^ mean (S.D)	4.16 (4.36), *n* = 32	3.68 (3.62), *n* = 28	0.36 (4.01), *n* = 28

EQ5-5D-5L score range: 0–1; PAM score range: 0–100; necessity score range: 5–25; concern score range: 5–25; overuse score range: 4–20; harm score range: 4–20.

a Attitudinal profiles at baseline based on the median cut-off score: acceptant (necessity >16, concern ≤12), ambivalent (necessity >16, concern >12), sceptic (necessity ≤16, concern >12) and indifferent (necessity ≤16, concern ≤12).

b Attitudinal profiles at week 24 based on the median cut-off score: acceptant (necessity >17, concern ≤13), ambivalent (necessity >17, concern >13), sceptic (necessity ≤17, concern >13) and indifferent (necessity ≤17, concern ≤13).

c NCD: necessity–concern difference.

∗ Abbreviations: BMQ, Beliefs about Medicines Questionnaire; EQ-5D-5L, EuroQol 5-Dimension 5-Level; IQR, interquartile range; NCD, necessity–concern difference; PAM, Patient Activation Measure; SD, standard deviation.

### Stakeholders’ perspectives

Ten stakeholders participated in the semi-structured interviews: rheumatology nurses (*n* = 4), rheumatologists (*n* = 3), and outpatient clinic management (*n* = 3). Thematic analysis of the interviews resulted in eight themes. Quotes reflecting the themes are shown in Table 4.

**Table 4:** Quotes supporting the respective themes.

(1) Maintaining quality of care
*‘If something goes wrong, then I’m the one who’s responsible. So I want to be sure things are going well. I’m afraid my patient might not be treated properly. That’s the underlying concern, really’*. (R1)
(2) Roles and responsibilities of healthcare providers
‘*If I am the lead practitioner, then I am ultimately responsible, but responsible for what, exactly? Because right now, I can’t see what’s happening [with the patient’s treatment]. I think that [final responsibility] needs to be very clearly arranged’*. (R2) ‘*Being a nurse involves a very different kind of training than that of doctors…Their background is more focused on nursing, and now they’re being expected to take on more medical responsibilities, so they need proper explanation for that’*. (R1)
(3) Suitability of patients
*‘I think it [suitability] is also about whether they [patients] … have some insight into their illness. Whether they’ll actually take action themselves if they notice something’s not right. Because it’s also important that they take some responsibility… People will also need to be at least somewhat technically capable, to understand things like pricking themselves and submitting the data’*. (N1) *‘Patients who are really eager to be actively involved in their health … People with good health literacy, but who also take pleasure in engaging with their health … People who like gadgets [would be suitable]’*. (M1)
(4) Organizational requirements
*‘In terms of resources, I think you need to have a solid network. You need reliable software where patients can access a platform in a very user-friendly way. Ideally, even with devices connected to the platform, so we’re not just manually retyping’*. (M2) *‘You need to make sure there’s reliable supply, otherwise everything falls apart. Also, you need a stable internet connection. People need to be able to contact us [healthcare providers], and if they [patients] can’t, then it won’t go well either’*. (M1)
(5) Potential added value
*‘I think this is one of the methods through which you can give patients insight, at a very low-intensity level, into the effects of their lifestyle. It allows you to really involve them more in their own treatment. As a result, fewer invasive treatment techniques may be required, and you can actually achieve better results and improved health outcomes’*. (M2) *‘I think colleagues also really enjoy being more involved with it. And as a nurse, you also take on a bit more personal responsibility. You’re allowed to say whether or not medication can be reduced, for example. And I know that many colleagues actually want to have more of that responsibility’*. (N1)
(6) Broader implementation and wider application of self-monitoring
‘*I think the people who don’t start urate-lowering therapy often experience so few symptoms from their gout that we [rheumatologists] don’t find it [self-monitoring] useful. So will those people remember if I told them a year ago, like, hey, you have … some kind of home measurement, rather than just … calling the hospital’?* (R1) *‘Research does show that when you refer gout patients back to primary care, it often ends up being managed less effectively. So I think that for patients we would normally refer back to, for example, the GP, self-monitoring could be a nice intermediate step to keep them under control’*. (R4)
(7) Support among healthcare providers
‘*If we can scientifically prove that this [self-monitoring] is superior [to usual care], then absolutely [I would use it]. But not by definition, because I just don’t know that yet. Let’s say: I’ll believe it when I see it’*. (M1) *‘I think that if you want to implement it [self-monitoring], you really need someone within the department who supports it and takes the lead to ensure that it actually gets implemented’*. (R4).
(8) System-level factors
*‘Especially in terms of reimbursement, I think it [self-monitoring] is very complex. At the moment, treatment is paid for in 9 out of 10 cases based on how often a patient sees the doctor. But the idea behind this process and protocol is that patients will see the doctor less frequently. So that requires some adjustments’*. (M2)

R: rheumatologist; N: nurse; M: outpatient clinic management. Numbers indicate individual participants.

#### Theme 1: Maintaining quality of care

Stakeholders emphasized that providing high-quality care to patients remains the foremost priority. Physicians expressed concerns about loss of control and decline in quality of care when shifting responsibilities to nurses. Reliable treatment and guidance by nurses is essential for the physicians’ confidence in the nurse-supported self-monitoring process.

#### Theme 2: Roles and responsibilities of healthcare providers

Stakeholders stressed that successful implementation of self-monitoring depends on clearly defined responsibilities and strong collaboration among HCPs. While nurses handle specific aspects of gout care, rheumatologists remain in the lead and should be kept involved in the patient’s treatment plan and have insight into their values. Trust in task reallocation relies on effective communication and clear protocols outlining responsibilities and safety measures.

Although initial challenges include resistance to change and limited digital skills, stakeholders observed that experience improves efficiency. Further task delegation (e.g. administrative tasks to doctors’ assistants) and staff training are key to adoption and building competence.

#### Theme 3: Suitability of patients

Self-monitoring is best suited to the ULT initiation phase, when frequent SU checks are needed. Patients should be independent and motivated and have adequate digital skills and health literacy. The younger, digitally competent patient who seeks autonomy and fewer hospital visits is deemed most suitable. Those preferring traditional care or lacking digital skills may be less suitable. Furthermore, a clear understanding of the purpose of self-monitoring is crucial. Poor fit of patient suitability for the intervention may lead to missed measurements, miscommunication, poor lifestyle choices, unnecessary use of resources and higher costs. Therefore, careful patient selection is essential despite being time-consuming.

##### Theme 4: Organizational requirement

Stakeholders noted that self-monitoring was well received by both HCPs and patients and, for some, required no further adjustments. However, success depends on meeting technical, logistical and organizational needs. A secure, integrated digital platform with automated messaging is essential but is currently lacking. Future apps should enable direct contact and personalization. POCT materials must be reliable and accessible, and digital tools, such as videos and chat, can support patients, while usual care remains necessary for those less suited to self-monitoring.

#### Theme 5: Potential added value

Stakeholders highlighted that self-monitoring adds significant value for patients and the healthcare system. For patients, it increases disease insight, encourages active participation, fosters a stronger sense of control and ownership, and potentially improves adherence to medication and lifestyle behaviour. It also allows for quicker, more frequent nurse contact and supports health outcomes comparable or superior to traditional care.

However, nurse time investment could be a barrier, and the initiation phase of ULT is time consuming, which may increase burden on patients and the system, but eases over time. Stakeholders noted that self-monitoring can make care pathways more efficient, lower costs and benefit the organization by serving a large number of patients and improving its public image. It also increases nurse job satisfaction through greater responsibility, more patient interaction and task variety.

#### Theme 6: Broader implementation and wider application of self-monitoring

Stakeholders predict increasing demand for self-monitoring due to healthcare pressures and a push towards prevention and self-management. Gout is particularly suited to nurse-supported self-monitoring given its chronic nature, straightforward clinical management and the clear relationship between SU levels and ULT dosage. While there is potential to expand implementation in both primary and secondary care, feasibility varies by patient group. Those on long-term or no ULT, or with infrequent gout flares, may lack motivation and forget to test. Some stakeholders question its benefit for these patients, though others value monitoring for tracking progression, promoting lifestyle changes and supporting adherence.

#### Theme 7: Support from healthcare providers for the intervention

This theme emphasizes that the demonstrated effectiveness and added value of the intervention are key to gaining HCPs’ support for self-monitoring. When these are evident, adoption increases. Enthusiasm from patients and providers can also motivate colleagues, and strong leadership helps reduce resistance and boost support.

#### Theme 8: System-level factors

There is a need for sustainable reimbursement models and compliance with legislation (e.g. the medical device regulation) for long-term viability. Successful implementation requires coordination among stakeholders such as health insurers, digital platform owners, suppliers and patients’ family members, who can support patients with their care.

## Discussion

This study investigated feasibility of nurse-supported SU self-monitoring for gout using a T2T approach. The intervention in the current setting was feasible and acceptable to patients and HCPs.

### Patient feasibility and perspectives

Patients were able to use POCT and the digital platform successfully, with most managing independently after watching an instructional video, and only a few needing extra support, confirming feasibility of the process. The intervention adherence was comparable to earlier studies, though selection bias and the younger age of participants, both associated with greater eHealth engagement, may have contributed [28–30]. Reminders by text and telephone likely further supported intervention adherence. Furthermore, the perceived ease of use was high, which is important as it supports continued use [31]. In this regard, patients expressed strong intent to continue self-monitoring, consistent with prior findings [16]. Nurse-supported self-monitoring achieved high patient acceptance, and patients reported that the messages from nurses, based on SU home measurements, helped them take medication as prescribed, improved perceived control over disease, which is consistent with previous research on nurse-led telemonitoring [16, 32]. However, 9% of participants did not complete the final questionnaire, which could be due to lower satisfaction. These participants however did continue self-measurements, suggesting they were reasonably engaged despite missing responses.

### Stakeholder feasibility and perspectives

Task reallocation to nurses was feasible, as rheumatologists were only minimally involved. Their role was limited to Ad hoc patient visits and responding to nurse queries, which was an important goal of the intervention. Due to this, nurses in particular experienced greater job satisfaction, consistent with evidence from chronic disease management [33]. Clear roles and responsibilities were highlighted as essential to avoid inferior care and team conflicts [34, 35] underscoring the importance of defined protocols.

Stakeholders noted benefits similar to those reported by patients for SU self-monitoring, including better outcomes, reduced burden on specialists and healthcare services, and lower costs [7]. However, views on wider implementation of nurse-supported self-monitoring were mixed, with stakeholders pointing to the need for evidence on added value and to barriers such as funding.

### Clinical effects

Regarding clinical outcomes, the overall SU target achievement rate (75%) aligns with secondary care standards [17]. However, ULT is lifelong, and short-term biochemical target attainment does not necessarily translate into sustained target attainment, long-term adherence, fewer flares or better patient outcomes. Because follow-up was limited to 24 weeks, this study only permits conclusions on short-term SU control within the context of a feasibility study. It must be noted, some participants had initiated ULT shortly before starting nurse-supported self-monitoring, resulting in lower study baseline SU values. Patient activation was modest at baseline and showed little change over 24 weeks, similar to other gout populations [36]. The mean patient activation score (PAM questionnaire) of participants was moderate (54). Nonetheless, this level of activation observed in a group that largely attained SU targets, reflecting effective engagement in nurse-supported self-monitoring. Beliefs about medication remained largely stable, but acceptant attitudes tended to decline while ambivalence slightly increased. These results underscore the importance of recognizing medication concerns, before as well as after start of ULT, and addressing these through ongoing support.

### Strengths and limitations

A key strength of this study is its comprehensive approach, combining patient and stakeholder perspectives, as well as short-term process and clinical outcomes. Semi-structured interviews provided in-depth insights from stakeholders, while data collection based on Bowen’s feasibility framework and the use of validated questionnaires ensured structure, reliability and comparability.

Generalizability might be limited due to the patient study population, which was relatively younger and well-educated, the latter being linked to better self-management [37, 38]. This selection likely favoured patients who are motivated, digitally literate and capable of adhering to complex self-management strategies. As such, the results may not be generalizable to the broader gout population, particularly those at highest risk of poor outcomes. Generalizability of findings may also be impaired due to the single-centre design, limited information on those who declined participation or dropped out and the lack of in-depth patient interviews. Finally, the healthcare resource utilization in this 24-week period appeared substantial with nurse involvement, and due to the nature of this study, we cannot yet conclude whether the total health care resources used are lower, comparable or higher than those in usual care, or alternative (nurse-led) gout management strategies.

### Clinical implications

Gout care was successfully shifted to nurses and patients taking a more active role. This approach may help manage rising patient volumes and improve care quality, provided that additional nursing tasks, such as sending patient reminders, can be automated to further reduce the workload. While feasibility was high, barriers remain. Future efforts should focus on integrating digital tools into nurse workflows, primarily by embedding self-monitoring data and communication within the patient’s EHR, enabling all tasks to be performed within a single central system. Patients expressed interest in more frequent monitoring, a view recognized by stakeholders, though this may lead to overuse and material waste. Still, more frequent testing could enhance adherence by boosting engagement, warranting further study on optimal testing frequency. Careful patient selection, favouring motivated, independent patients with adequate or developable digital skills and health literacy, is crucial, and interventions to improve eHealth literacy may enhance effectiveness for those currently lacking these skills [39]. Furthermore, evidence on long-term effectiveness on SU outcomes and time savings is needed, as well as an exploration of sustainable reimbursement models. This hybrid care model shows particular promise for primary care and regions with limited access or resources, and therefore, future directives should explore whether general practices can deliver this care.

## Conclusion

Nurse-supported T2T self-monitoring of SU in rheumatological care achieved high patient acceptance and adherence and was practical in this selected group of gout patients. Patients reported feelings of improved disease control, nurses experienced greater job satisfaction, and stakeholders perceived potential benefits in terms of outcomes and costs, though these could not be formally evaluated in this study. Short-term adherence to home measurements was high and SU target attainment at 24 weeks was encouraging. Given the selected study population, absence of a control group and short follow-up duration, larger comparative randomized studies with longer follow-up and formal economic evaluation are warranted to evaluate (cost-)effectiveness.

## Supplementary Material

rkag064_Supplementary_Data

## Data Availability

The data underlying this article will be shared on reasonable request to the corresponding author.

## References

[1] Global, regional, and national burden of gout, 1990-2020, and projections to 2050: a systematic analysis of the Global Burden of Disease Study 2021. Lancet Rheumatol 2024;6:e507–17. 38996590 10.1016/S2665-9913(24)00117-6 PMC11263476

[2] Punjwani S , Jani C, Liu W et al Burden of gout among different WHO regions, 1990-2019: estimates from the global burden of disease study. Sci Rep 2024;14:15953. 38987583 10.1038/s41598-024-61616-z PMC11236997

[3] Dalbeth N , Gosling AL, Gaffo A, Abhishek A. Gout. Lancet 2021;397:1843–55. 33798500 10.1016/S0140-6736(21)00569-9

[4] Perez-Ruiz F , Moreno-Lledó A, Urionagüena I, Dickson AJ. Treat to target in gout. Rheumatology (Oxford) 2018;57:i20–6. 29272512 10.1093/rheumatology/kex442

[5] Richette P , Doherty M, Pascual E et al 2016 updated EULAR evidence-based recommendations for the management of gout. Ann Rheum Dis 2017;76:29–42. 27457514 10.1136/annrheumdis-2016-209707

[6] Dehlin M , Jacobsson L, Roddy E. Global epidemiology of gout: prevalence, incidence, treatment patterns and risk factors. Nat Rev Rheumatol 2020;16:380–90. 32541923 10.1038/s41584-020-0441-1

[7] Doherty M , Jenkins W, Richardson H et al Efficacy and cost-effectiveness of nurse-led care involving education and engagement of patients and a treat-to-target urate-lowering strategy versus usual care for gout: a randomised controlled trial. Lancet 2018;392:1403–12. 30343856 10.1016/S0140-6736(18)32158-5 PMC6196879

[8] Riches PL , Alexander D, Hauser B, Kuske B, Krause A. Evaluation of supported self-management in gout (GoutSMART): a randomised controlled feasibility trial. Lancet Rheumatol 2022;4:e320–8. 38294032 10.1016/S2665-9913(22)00062-5

[9] Russell MD , Roddy E, Rutherford AI et al Treat-to-target urate-lowering therapy and hospitalizations for gout: results from a nationwide cohort study in England. Rheumatology (Oxford) 2023;62:2426–34. 36355461 10.1093/rheumatology/keac638 PMC10321109

[10] NICE. Evidence review for the best serum urate level target to use when treating-to-target in gout. 2022. https://www.ncbi.nlm.nih.gov/books/NBK589580/ (16 Novemer 2025, date last accessed). 36921075

[11] Rees F , Jenkins W, Doherty M. Patients with gout adhere to curative treatment if informed appropriately: proof-of-concept observational study. Ann Rheum Dis 2013;72:826–30. 22679303 10.1136/annrheumdis-2012-201676

[12] Mikuls TR , Cheetham TC, Levy GD et al Adherence and outcomes with urate-lowering therapy: a site-randomized trial. Am J Med 2019;132:354–61. 30503879 10.1016/j.amjmed.2018.11.011 PMC6399023

[13] Boniol M , Kunjumen T, Nair TS et al The global health workforce stock and distribution in 2020 and 2030: a threat to equity and 'universal’ health coverage? BMJ Glob Health 2022;7:3–5. doi:10.1136/bmjgh-2022-009316. PMC9237893 35760437

[14] Riches PL , Sing K, Berg K. Point-of-care uric acid testing is useful in routine clinical care of gout. Arthritis Res Ther 2019;21:117. 31072349 10.1186/s13075-019-1891-1 PMC6509753

[15] Paraskos J , Berke Z, Cook J et al An analytical comparison between point-of-care uric acid testing meters. Expert Rev Mol Diagn 2016;16:373–82. 26689648 10.1586/14737159.2016.1134326

[16] Michael TJF , Chan JS, Hughes S et al The experiences and perspectives of people with gout on urate self-monitoring. Health Expect 2024;27:e14071. 38742836 10.1111/hex.14071 PMC11092534

[17] van der Ven J , Flendrie M, van Dijck F et al Cost-effectiveness of nurse-led home monitoring of serum urate for gout patients starting with urate-lowering therapy in secondary care: a modeling study. Semin Arthritis Rheum 2025;74:152782. 40763440 10.1016/j.semarthrit.2025.152782

[18] Bowen DJ , Kreuter M, Spring B et al How we design feasibility studies. Am J Prev Med 2009;36:452–7. 19362699 10.1016/j.amepre.2009.02.002 PMC2859314

[19] Lancaster GA , Dodd S, Williamson PR. Design and analysis of pilot studies: recommendations for good practice. J Eval Clin Pract 2004;10:307–12. 15189396 10.1111/j..2002.384.doc.x

[20] Gaffo AL , Dalbeth N, Saag KG et al Brief report: validation of a definition of flare in patients with established gout. Arthritis Rheumatol 2018;70:462–7. 29161469 10.1002/art.40381

[21] Duong TV , Nguyen TTP, Pham KM et al Validation of the Short-Form Health Literacy Questionnaire (HLS-SF12) and its determinants among people living in rural areas in Vietnam. Int J Environ Res Public Health 2019;16:3346. doi:10.3390/ijerph16183346. 31514271 PMC6765800

[22] Pharos. Quickscan digitale vaardigheden. 2024. Available from: https://www.pharos.nl/wp-content/uploads/2024/08/Quickscan_digitale-vaardigheden_082024.pdf

[23] Ensink CJ , Keijsers NL, Groen BE. Translation and validation of the system usability scale to a Dutch version: D-SUS. Disabil Rehabil 2024;46:395–400. 36573399 10.1080/09638288.2022.2160837

[24] Herdman M , Gudex C, Lloyd A et al Development and preliminary testing of the new five-level version of EQ-5D (EQ-5D-5L). Qual Life Res 2011;20:1727–36. 21479777 10.1007/s11136-011-9903-x PMC3220807

[25] Horne R , Weinman J, Hankins M. The beliefs about medicines questionnaire: the development and evaluation of a new method for assessing the cognitive representation of medication. Psychol Health 1999;14:1–24.

[26] van Geffen ECG , Philbert D, van Boheemen C et al Patients’ satisfaction with information and experiences with counseling on cardiovascular medication received at the pharmacy. Patient Educ Couns 2011;83:303–9. 21550196 10.1016/j.pec.2011.04.004

[27] Braun V , Clarke V. Using thematic analysis in psychology. Qual Res Psychol 2006;3:77–101.

[28] Cea Soriano L , Rothenbacher D, Choi HK, García Rodríguez LA. Contemporary epidemiology of gout in the UK general population. Arthritis Res Ther 2011;13:R39. 21371293 10.1186/ar3272 PMC3132018

[29] Vazquez CE , Mauldin RL, Mitchell DN, Ohri F. Sociodemographic factors associated with using eHealth for information seeking in the United States: cross-sectional population-based study with 3 time points using health information national trends survey data. J Med Internet Res 2024;26:e54745. 39141905 10.2196/54745 PMC11358649

[30] Michael TJF , Wright DFB, Chan JS et al Patient-led urate self-monitoring to improve clinical outcomes in people with gout: a feasibility study. ACR Open Rheumatol 2024;6:403–11. 38591107 10.1002/acr2.11666 PMC11246832

[31] Ammenwerth E. Technology acceptance models in health informatics: TAM and UTAUT. Stud Health Technol Inform 2019;263:64–71. 31411153 10.3233/SHTI190111

[32] Gordon K , Dainty KN, Steele Gray C et al Experiences of complex patients with telemonitoring in a nurse-led model of care: multimethod feasibility study. JMIR Nursing 2020;3:e22118. 34406972 10.2196/22118 PMC8408315

[33] Stephen C , McInnes S, Halcomb E. The feasibility and acceptability of nurse-led chronic disease management interventions in primary care: an integrative review. J Adv Nurs 2018;74:279–88. 28880393 10.1111/jan.13450

[34] Brault I , Kilpatrick K, D’Amour D et al Role clarification processes for better integration of nurse practitioners into primary healthcare teams: a multiple-case study. Nurs Res Pract 2014;2014:170514. 25692039 10.1155/2014/170514 PMC4322308

[35] Martínez-González NA , Tandjung R, Djalali S, Rosemann T. The impact of physician-nurse task shifting in primary care on the course of disease: a systematic review. Hum Resour Health 2015;13:55. 26149447 10.1186/s12960-015-0049-8 PMC4493821

[36] Coburn BW , Bendlin KA, Sayles H et al Allopurinol medication adherence as a mediator of optimal outcomes in gout management. J Clin Rheumatol 2017;23:317–23. 28816767 10.1097/RHU.0000000000000561

[37] Geboers B , de Winter AF, Spoorenberg SL, Wynia K, Reijneveld SA. The association between health literacy and self-management abilities in adults aged 75 and older, and its moderators. Qual Life Res 2016;25:2869–77. 27101999 10.1007/s11136-016-1298-2 PMC5065597

[38] Kim K , Yang Y, Wang Z et al A systematic review of the association between health literacy and pain self-management. Patient Educ Couns 2022;105:1427–40. 34629232 10.1016/j.pec.2021.09.037

[39] Qian J , Yao X, Liu T. Assessment of electronic health literacy and its association with self-management among gout patients: a cross-sectional study. Arch Rheumatol 2024;39:358–67. 39507850 10.46497/ArchRheumatol.2024.10397 PMC11537690

