# Supplementary material for: Nurse-supported self-monitoring of serum urate by gout patients using a treat-to-target approach: a feasibility study
Source: Rheumatol Adv Pract. 2026 Jun 5;10(3):rkag064. doi: 10.1093/rap/rkag064 (PMC13303286; doi:10.1093/rap/rkag064)
Supplement: rkag064_Supplementary_Data [file rkag064_supplementary_data.zip › Supplementary Material 3.docx]

**Supplementary Material 3: Results**

*Table S1: Patient experience with digital technologies (n = 32).*

| Characteristics | Yes | No | With help from others |
| --- | --- | --- | --- |
| Owns a laptop, smartphone, or tablet, n (%) | 32 (100) | 0 (0) |  |
| Searches health information on the internet, n (%) | 28 (88) | 4 (12) | 0 (0) |
| Uses email, n (%) | 31 (97) | 0 (0) | 1 (3) |
| Uses apps, n (%) | 31 (97) | 0 (0) | 1 (3) |
| Downloads apps, n (%) | 30 (94) | 2 (6) | 0 (0) |
| Uses DigiD, n (%) | 31 (97) | 0 (0) | 1 (3) |

*Table S2: Satisfaction with home monitoring and 1 – 5 star rating from the 12 week (n = 31) and 24 week (n = 29) questionnaire.*

| Satisfaction | Week 12 | Week 24 |
| --- | --- | --- |
| I would not recommend home monitoring n(%) | 0 (0) | 0 (0) |
| I would recommend home monitoring to a few people n(%) | 2 (6.5) | 3 (10.3) |
| I would recommend home monitoring to many people n(%) | 17 (54.8) | 15 (51.7) |
| I would recommend home monitoring to everybody n(%) | 12 (38.7) | 11 (37.9) |
| ★ n(%) | 0 (0) | 0 (0) |
| ★★ n(%) | 0 (0) | 0 (0) |
| ★★★ n(%) | 4 (12.9) | 1 (3.4) |
| ★★★★ n(%) | 15 (48.4) | 20 (69) |
| ★★★★★ n(%) | 12 (38.7) | 8 (27.6) |

*Table S3: Device attractiveness statements from the 12 week (n = 31) and 24 week (n = 29) questionnaire.*

| Attractiveness device | Week 12 | Week 24 |
| --- | --- | --- |
| Unattractive, n(%) | 0 (0) | 0 (0) |
| Poor, n(%) | 1 (3.2) | 0 (0) |
| Neutral, n(%) | 23 (74.2) | 20 (69) |
| Attractive, n(%) | 5 (16.1) | 8 (27.6) |
| Very attractive, n(%) | 2 (6.5) | 1 (3.4) |

*Table S4: Device appropriateness statements from the 12 week (n = 31) and 24 week (n = 29) questionnaire.*

| Appropriateness | Week 12 | Week 24 |
| --- | --- | --- |
| Totally inappropriate n(%) | 0 (0) | 1 (3.1) |
| Mostly inappropriate n(%) | 1 (3.1) | 0 (0) |
| Acceptable n(%) | 5 (15.6) | 5 (15.6) |
| Appropriate n(%) | 7 (21.9) | 7 (21.9) |
| Very appropriate n(%) | 18 (56.2) | 16 (50) |

*Table S5: Future demand and intention to use statements with regard to home monitoring from the 12 (n = 31) and 24 week (n = 29) questionnaire.*

| Future demand | Week 12 | Week 24 |
| --- | --- | --- |
| I would like to use home monitoring in the future, n(%) |  |  |
| Totally agree | 12 (38.7) | 13 (44.8) |
| Agree | 18 (58.1) | 15 (51.7) |
| Neutral | 1 (3.2) | 1 (3.4) |
| Disagree | 0 (0) | 0 (0) |
| Totally disagree | 0 (0) | 0 (0) |
| In the future, I would use home monitoring, n(%) |  |  |
| Much more often | 4 (12.9) | 4 (13.8) |
| More often | 11 (35.5) | 7 (24.1) |
| Equally often | 16 (51.6) | 15 (51.7) |
| Less often | 0 (0) | 3 (10.3) |
| I would not use it anymore | 0 (0) | 0 (0) |
| I would pay for home monitoring, n(%) |  |  |
| Totally agree | 3 (9.7) | 4 (13.8) |
| Agree | 8 (25.8) | 11 (37.9) |
| Neutral | 14 (45.2) | 10 (34.5) |
| Disagree | 3 (9.7) | 3 (10.3) |
| Totally disagree | 3 (9.7) | 1 (3.4) |

*Table S6: Doses of ULT in milligrams at baseline and 24 weeks, stratified by two different SU targets. N" indicates the total number of patients per SU target group; "n" refers to the number of patients receiving medication within that group.*

|  | Baseline | 24 weeks |
| --- | --- | --- |
| Target 0.36 mmol/L, N = 17 |  |  |
| Allopurinol, mean (SD), n = 16 | 134 (70) | 325 (117) |
| Febuxostat, n = 1 | 40 | 80 |
| Target 0.30 mmol/L, N = 15 |  |  |
| Allopurinol (mg), mean (SD), n = 13 | 254 (180) | 450 (196) |
| Febuxostat (mg), n = 1 | 120 | 120 |
| Benzbromaron (mg), n = 1 | 50 | 50 |

For patients in the 0.30 group, the median allopurinol dose increased from 100 mg (IQR: 100–300) at baseline to 400 mg (IQR: 300–600) at 24 weeks. In the 0.36 group, the median dose increased from 100 mg (IQR: 100–112.5) at baseline to 300 mg (IQR: 200–400) at 24 weeks.

*Supplementary Table S7: List of (serious) adverse events reported by patients in the 4-weekly questionnaires. % Patients shows the percentage of patients experiencing the adverse event for their respective drug category.*

**Adverse Events**

| **Drug** | **Organ system** | **# Patients** | **% Patients** |
| --- | --- | --- | --- |
| **Allopurinol** | Digestive system | 9 | 31% |
|  | Skin | 4 | 4% |
|  | Musculoskeletal | 7 | 24% |
|  | Neurological | 1 | 4% |
|  | General / Other (e.g. fatigue, flushing, excessive (night) sweating | 5 | 17% |
|  | Cardiovascular | 1 | 3% |
| **Benzbromaron** | Digestive system | 1 | 100% |
|  | Musculoskeletal | 1 | 100% |
| **Febuxostat** | Musculoskeletal | 2 | 100% |

**Serious adverse events**

One serious adverse event (SAE) was reported: a hospital admission related to a knee prosthesis on 2024-06-07

**Supplementary Text S1: Additional information on practicality**

Patient-reported feedback and suggestions on practical aspects of the intervention was collected from the questionnaires at both week 12 and week 24. Three patients found the video unclear regarding the application of the blood drop. Patient preferences included more personal contact early in treatment (n = 2), app-based question submission (n = 2), shorter POCT instructions (n = 1), in-app notifications (n = 1), integrated medication information (n = 1), laboratory results within the app (n = 1), and centralized digital information (n = 1). Other reported issues included difficulties with medication ordering (n = 2), app notifications (n = 2), the relation between the POCT-device and laboratory results (n = 1), and long-term follow-up (n = 1). In addition, 8 patients themselves reported difficulties with therapy adherence.

**Supplementary Text S2: Additional information on clinical process outcomes**

Reasons for 19 rheumatologist visits were as following: 3 for gout flares, 9 for examinations or diagnostic discussions, 1 for therapy non-adherence, and 6 routine visits without clear indication.

Questions forwarded by nurses to rheumatologists mainly concerned prescriptions (n=10), gout flare management (n=9), and urate-lowering therapy use or dosing (n=8). 4 were on side effects, 1 on a contraindication, and 1 on self-measurement frequency.


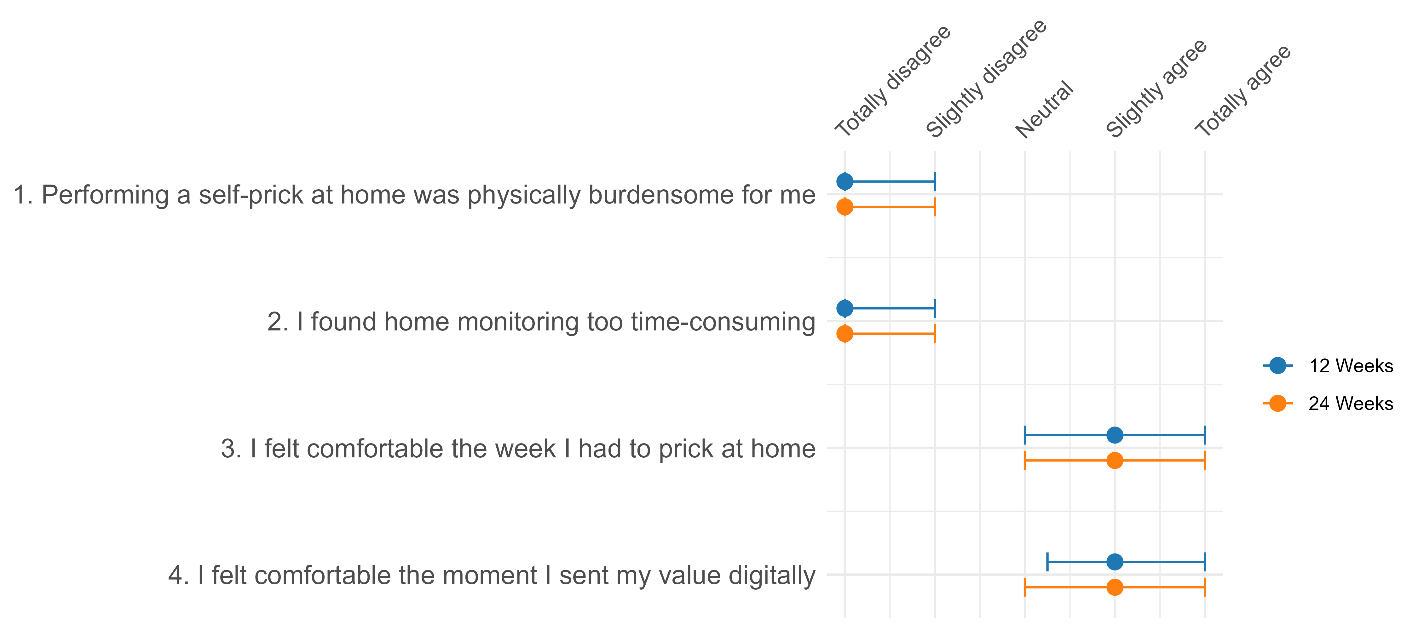


**Figure S1. Patient burden of the intervention.** At week 12, n = 29, 31, 28, and 30, and at week 24, n = 29, 29, 25, and 25, for questions 1–4, respectively. Dots represent the median on a 5-point Likert scale, and bars indicate the first and third quartile. Participants could also select 'not applicable'; these responses were excluded from the sample size.


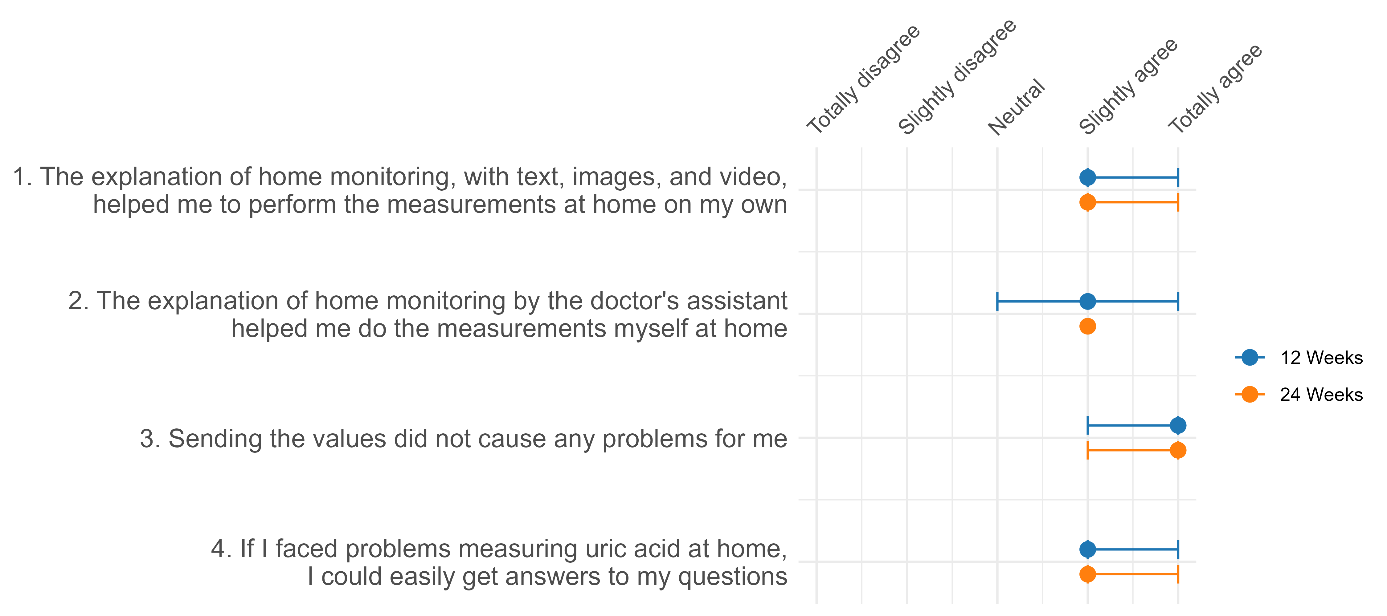


**Figure S2. Practicality of the intervention.** At week 12 (n=31,18,31,25) and at week 24 (n=29,16,29,24). Dots represent the median on a 5-point Likert scale, and bars indicate the first and third quartile. Participants could also select 'not applicable'; these responses were excluded from the sample size.


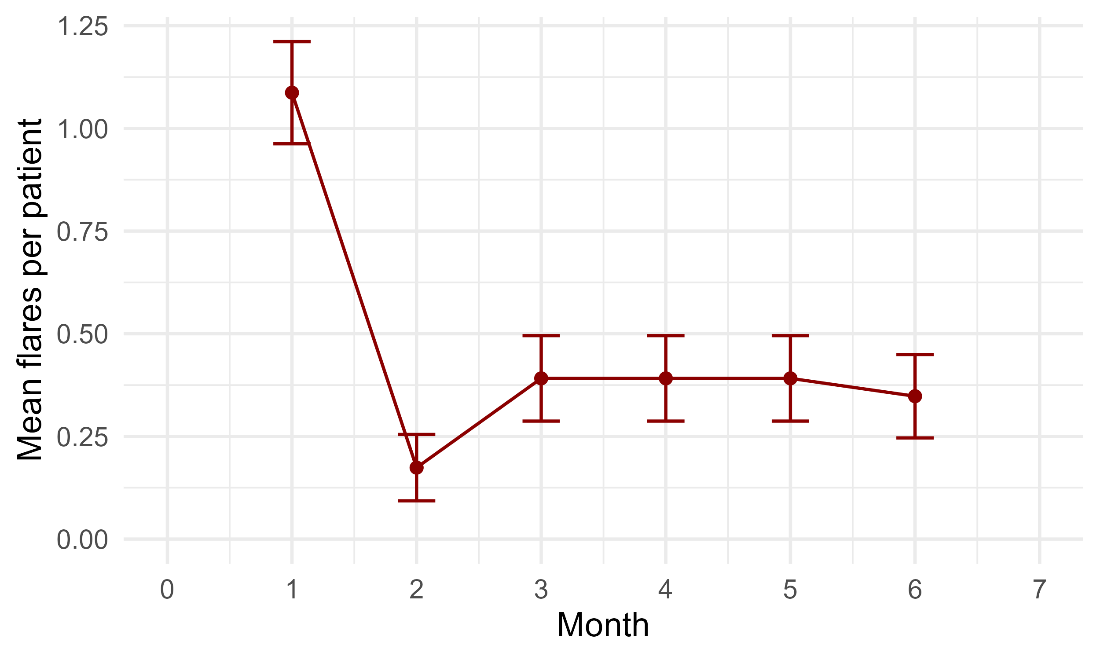


**Figure S3.** **Patient-reported gout flare rate during the study period.** Months are shown on the x-axis and mean per patient per month gout flare rate on the y-axis. Error bars show standard error.
