# Supplementary material for: Nurse-supported self-monitoring of serum urate by gout patients using a treat-to-target approach: a feasibility study
Source: Rheumatol Adv Pract. 2026 Jun 5;10(3):rkag064. doi: 10.1093/rap/rkag064 (PMC13303286; doi:10.1093/rap/rkag064)
Supplement: rkag064_Supplementary_Data [file rkag064_supplementary_data.zip › 25-283 Supplementary Data S2.docx]

**Supplementary Data S2: Methods (questionnaires & Interview guide)**

Section 1. General Information and Experience with Digital Applications

1. What is your marital status?

☐ Married (including registered partnership)

☐ Divorced

☐ Widowed

☐ Never been married

2. What is your highest level of completed education?

☐ No education (did not complete primary school)

☐ Primary education

☐ Lower secondary vocational education

☐ Lower secondary general education

☐ Upper secondary vocational education

☐ Upper secondary general education

☐ Higher professional education

☐ University education

☐ Other: _________________________

3. Which situation applies most to you?

☐ I am employed (paid work), 32 hours or more per week

☐ I am employed (paid work), 20 or more but less than 32 hours per week

☐ I am employed (paid work), 12 or more but less than 20 hours per week

☐ I am employed (paid work), less than 12 hours per week

☐ I am (early) retired (state pension, early retirement scheme)

☐ I am unemployed / looking for work (registered with the employment office)

☐ I am occupationally disabled

☐ I receive social assistance benefits

☐ I am a full-time housewife/husband

☐ Studying

Section 2. Evaluation of digital skills based on Pharos Quickscan

4. Do you have access to a computer, smartphone or tablet with internet?

☐ Yes

☐ No

5. Do you search for information (e.g. about health or illness) online?

☐ Yes

☐ Yes, with help from others

☐ No

6. Do you use email?

☐ Yes

☐ Yes, with help from others

☐ No

7. Do you use apps?

☐ Yes

☐ Yes, with help from others

☐ No

8. Can you download an app yourself?

☐ Yes

☐ Yes, with help from others

☐ No

9. Do you use DigiD for banking, insurance, or healthcare?

☐ Yes

☐ Yes, with help from others

☐ No

Section 3. Experience with the patient portal and video consultations

1. How much experience do you have with MijnMaartenskliniek?

☐ None

☐ Little

☐ Average

☐ Much

☐ Very much

2. How much experience do you have with video calling with a healthcare provider?

☐ None

☐ Little

☐ Average

☐ Much

☐ Very much

Section 4. Health literacy

*Short-form health literacy survey tool.*

*Answer using:*

☐ Very easy ☐ Easy ☐ Difficult ☐ Very difficult

1. How easy or difficult is it for you to find information about illnesses and treatments that are

relevant to you?

2. How easy or difficult is it for you to understand the information in a medicine leaflet?

3. How easy or difficult is it for you to weigh the pros and cons of different treatment options?

4. How easy or difficult is it for you to find information about how to manage your mental health

(e.g., stress)?

5. How easy or difficult is it for you to find activities that are good for your (mental) health?

6. How easy or difficult is it for you to understand information in media (e.g., internet, TV,

newspapers) about healthy living?

7. How easy or difficult is it for you to judge which everyday actions (eating, drinking, exercising)

affect your health?

8. How easy or difficult is it for you to call an ambulance in an emergency?

9. How easy or difficult is it for you to understand why you need health checks (e.g., blood sugar, blood pressure)?

10. How easy or difficult is it for you to judge which vaccinations you might need?

11. How easy or difficult is it for you to protect yourself from illness based on advice from family or friends?

12. How easy or difficult is it for you to join a sports club or activity class?

Section 5. Ease of Use of the Home Meter

*System Usability Scale (SUS).*

Answer using:

☐ Totally agree ☐ Slightly Agree ☐ Neutral ☐ Slightly Disagree ☐ Totally Disagree

1. I think I would like to use the home meter frequently.

2. I found the home meter unnecessarily complex.

3. I thought the home meter was easy to use.

4. I think I would need the support of a technical person to use the home meter.

5. I found the various functions of the home meter well integrated.

6. I think there were too many inconsistencies in the home meter.

7. I imagine that most people would learn to use the home meter very quickly.

8. I found the home meter very cumbersome to use.

9. I felt very confident using the home meter.

10. I needed to learn a lot of things before I could get going with the home meter.

Section 6. Attractiveness and appropriateness of the home monitor

1. How attractive is the design of the home monitor?

☐ Very unattractive – bad design, unpleasant to look at, poor color combination

☐ Poor – dull or inconsistent design, weak use of color

☐ Neutral – average, neither attractive nor unattractive

☐ Attractive – consistent and professional design

☐ Very attractive – visually appealing, memorable, stands out, color enhances functionality

2. Is the home monitor appropriate for people with gout (in appearance, language, and design)?

☐ Totally inappropriate – unclear or confusing for the target group

☐ Mostly inappropriate – often unclear or confusing for the target group

☐ Acceptable – not specifically designed for the target group, may sometimes be unclear or confusing

☐ Appropriate – suitable for the target group, with minor issues

☐ Very appropriate – clear, well-designed, and tailored for people with gout

Section 7. Usefulness of Home Monitoring

Answer using:

☐ Totally agree ☐ Slightly Agree ☐ Neutral ☐ Slightly Disagree ☐ Totally Disagree

1. Home monitoring allows me to have more insight into my uric acid levels

2. The messages from the nurse based on the home measurements help me take my medication

correctly

3. I like the idea of a nurse viewing and checking my self-measured values through the app

4. I like having a nurse advising me on my (dose of) medication through the app

5. Home monitoring makes me feel more involved in my treatment

6. I find home monitoring a useful addition to my care

7. Home monitoring helps me better manage my gout better

8. Home monitoring ensures that I experience more control over the treatment of my gout

9. I think my gout symptoms have decreased due to home monitoring

Section 8. Satisfaction with home monitoring

1. Would you recommend home monitoring to others?

☐ To anyone

☐ To many people

☐ To a few people

☐ To no one

2. Star rating of home monitoring

☐ ★

☐ ★★

☐ ★★★

☐ ★★★★

☐ ★★★★★

Section 9. Demand for home monitoring

1. I want to continue home monitoring in the future

☐ Totally agree

☐ Slightly Agree

☐ Neutral

☐ Slightly disagree

☐ Totally disagree

2. In the future I would:

☐ Much more often

☐ More often

☐ Equally often

☐ Less often

☐ I would not use it anymore

3. If the home meter wasn't free, I would be willing to pay for it

☐ Totally agree

☐ Slightly Agree

☐ Neutral

☐ Slightly disagree

☐ Totally disagree

4. How often did you use the home meter recently?

☐ Only at scheduled times (every 4 weeks)

☐ More than scheduled

☐ Less than scheduled

5. Have you missed a measurement in the last 24 weeks?

☐ Yes, because: ____________________________

☐ No

Section 10. Practical matters and perceived burden of home monitoring

Answer using:

☐ Totally agree ☐ Slightly Agree ☐ Neutral ☐ Slightly Disagree ☐ Totally Disagree ☐ Not applicable

1. The explanation of the device, with text, images, and video helped me to perform the

measurements at home on my own

2. The explanation of the device by the doctor’s assistant helped me do the measurements

myself at home

3. Sending the values did not cause any problems for me

4. If I faced problems measuring uric acid at home, I could easily get answers to my questions

5. Performing a self-prick at home was physically burdensome for me

6. I found home monitoring too time-consuming

7. I felt comfortable during the week I had to prick at home

8. I felt comfortable the moment I sent my value digitally

Section 11. How actively you manage your health and illness

Patient Activation Measure (PAM-13)

Answer using:

☐ Totally agree ☐ Slightly Agree ☐ Neutral ☐ Slightly Disagree ☐ Totally Disagree ☐ Not applicable

1. Ultimately, I am responsible for my own health.

2. Taking an active role in my health care has the biggest impact on my health.

3. I am confident that I can help prevent or reduce health problems.

4. I know what each of my prescribed medications is for.

5. I am confident that I can judge when I need to see a doctor or when I can handle a health

problem myself.

6. I am confident that I can tell a doctor about my concerns, even if they don’t ask.

7. I am confident that I can perform medical tasks at home when necessary.

8. I understand my health problems and what causes them.

9. I know what treatments are available for my health conditions.

10. I have been able to maintain changes in my lifestyle (such as healthy eating or exercise).

11. I know how to prevent health problems.

12. I am confident that I can come up with solutions to new health problems.

13. I am confident that I can maintain lifestyle changes (such as healthy eating and exercise),

even during stressful times.

Section 12. Your Health Today

EQ-5D-5L

Please select one option per category:

**Mobility**

☐ I have no problems walking

☐ I have slight problems walking

☐ I have moderate problems walking

☐ I have severe problems walking

☐ I am unable to walk

**Self-care**

☐ I have no problems washing or dressing myself

☐ I have slight problems washing or dressing myself

☐ I have moderate problems washing or dressing myself

☐ I have severe problems washing or dressing myself

☐ I am unable to wash or dress myself

**Usual activities**

*(e.g., work, study, household tasks, family or leisure activities)*

☐ I have no problems doing my usual activities

☐ I have slight problems doing my usual activities

☐ I have moderate problems doing my usual activities

☐ I have severe problems doing my usual activities

☐ I am unable to do my usual activities

**Pain / discomfort**

☐ I have no pain or discomfort

☐ I have slight pain or discomfort

☐ I have moderate pain or discomfort

☐ I have severe pain or discomfort

☐ I have extreme pain or discomfort

**Anxiety/depression**

☐ I am not anxious or depressed

☐ I am slightly anxious or depressed

☐ I am moderately anxious or depressed

☐ I am severely anxious or depressed

☐ I am extremely anxious or depressed

**Visual Analogue Scale (VAS)**

We would like to know how good or bad your health is TODAY.

This scale ranges from 0 to 100:

• 100 means the best health you can imagine

• 0 means the worst health you can imagine

Please mark an “X” on the scale below to indicate how your health is TODAY, and write the number in the box.

|-------------------------------------------------------------------------------------------------------------------------------------|

0 100

Worst imaginable health Best imaginable health

My health today (0–100): _______

Section 13. Beliefs About Medicines

Beliefs About Medicines Questionnaire (BMQ)

**Your Thoughts on the Medicines Prescribed to You**

*Answer using:*

☐ Totally agree ☐ Slightly Agree ☐ Neutral ☐ Slightly Disagree ☐ Totally Disagree

1. At the moment, my health depends on my medicines.

2. I worry about having to take medicines.

3. My life would be very difficult without my medicines.

4. Sometimes I worry about the long-term effects of my medicines.

5. Without my medicines, I would become very ill.

6. I don’t know enough about what my medicines do.

7. My future health depends on my medicines.

8. My medicines disrupt my life.

9. Sometimes I’m afraid I’ll become too dependent on my medicines.

10. My medicines prevent my condition from getting worse.

11. These medicines have unpleasant side effects.

**Your Thoughts on Medicines in General**

*Answer using:*

☐ Totally agree ☐ Slightly Agree ☐ Neutral ☐ Slightly Disagree ☐ Totally Disagree

12. Doctors prescribe too many medicines.

13. People who take medicines should stop their treatment from time to time.

14. Most medicines are addictive.

15. Natural remedies are safer than medicines.

16. Medicines do more harm than good.

17. All medicines are poison.

18. Doctors rely too much on medicines.

19. If doctors had more time for their patients, they would prescribe fewer medicines.

Section 14. Hospital & Emergency Care

1. Have you been hospitalized since the previous questionnaire?

☐ Yes

☐ No

2. Have you visited the emergency department or GP out-of-hours service?

☐ Yes

☐ No

Section 15. Additional Comments

Do you have any other comments or concerns about the home meter or the entire home monitoring

process?

**Stakeholder Interviewguide**

The interview guide that was used for the stakeholder interviews can be found below.

To begin, I would first like to ask you a few general questions.

- What is your age?
- What is your position?
  o How long have you been in this position?
- How often do you see patients with gout? (daily/weekly/monthly/yearly)

1. What is your experience with home monitoring of uric acid for patients with gout?

Acceptability:

2. Do you consider home monitoring of uric acid to be appropriate?

- For patients with gout?
  i. And why do you think so?
  ii. What positive and negative effects do you think home monitoring has for patients with gout?
- For yourself in your own role within the hospital? (appropriateness within your scope of duties)
  i. And why do you think so?
  ii. In which situations yes or no?
  iii. What positive and negative effects do you think home monitoring has for you?
- For the Sint Maartenskliniek as an organization?
  i. What makes you say that?

3. To what extent would you as a doctor/nurse/project leader be willing to use home monitoring in the future (in other words, are you open to it?)

- Why or why not?
- What factors play a role in your decision whether or not to use home monitoring in practice? And why?
  i. Does reimbursement play a role?
  ii. Does time investment play a role? (i.e., how much time it takes you)
  iii. Does inclusion or exclusion of home monitoring in clinical guidelines play a role?
  iv. Does the need for additional training play a role?
  v. Does the level of (digital) self-management skills of the patient play a role?

Demand:

4. Do you personally feel a need for home monitoring for patients using urate-lowering therapy?

- And why?
- To what extent do you expect a change in this need in the future?
  i. Does this also apply to patients who are not using urate-lowering medication?
  1. Why or why not?
- How do you expect support among colleagues for implementing home monitoring in clinical practice?
  i. Why do you expect that?
  ii. What influences this support?
  1. To what extent does prioritization of home monitoring over other initiatives within your field play a role?
  2. Whether or not leadership is involved?
  3. Whether or not support and/or training is available?
- (For health insurers) And to what extent is there a societal need for home monitoring of uric acid in people with gout who use ULT?

5. Are there factors that could influence the need for home monitoring in people with gout? *(if time permits)*

- Increase it?
- Decrease it?

Implementation / Integration / Practicality

6. To what extent would you consider home monitoring to be successful?

- Why or why not?
- What were the success factors?
  i. From the perspective of doctors/nurses/project leaders
- And what were the barriers/obstacles?
  i. From the perspective of doctors/nurses/project leaders

7. What steps are necessary (or what needs to change) to make home monitoring feasible in practice?

- What resources are needed?
  i. Time
  ii. Money
  iii. Personnel
  iv. Education
- How do you view these resources (positive/negative)? Why?
- Which stakeholders are needed?
- And what changes in current practice within our hospital are necessary?
  i. And what changes are needed outside the hospital?
  1. Reimbursement?
  2. Legislation?
- Are any changes needed to the device itself? If so, which?

8. Could home monitoring be used more broadly within secondary care for patients with gout?

- For flares and side effects?
- For a broader patient population?
  i. For example, not only patients who are just starting long-term urate-lowering therapy?
